# The Therapeutic Effect of Cytokine-Induced Killer Cells on Pancreatic Cancer Enhanced by Dendritic Cells Pulsed with K-Ras Mutant Peptide

**DOI:** 10.1155/2011/649359

**Published:** 2011-10-19

**Authors:** Guang Tan, Xin Zhang, Hongbo Feng, Haifeng Luo, Zhongyu Wang

**Affiliations:** Department of General Surgery, The First Affiliated Hospital of Dalian Medical University, No. 222, Zhongshan Road, Dalian 116011, China

## Abstract

*Objective*. This study is to investigate the role of the CIKs cocultured with K-ras-DCs in killing of pancreatic cancer cell lines, PANC-1 (K-ras^+^) and SW1990 (K-ras^−^). *Methods*. CIKs induced by IFN-*γ*, IL-2, and anti-CD3 monoantibody, K-ras-DCCIKs obtained by cocultivation of k-ras-DCs and CIKs. Surface markers examined by FACS. IFN-*γ* IL-12 ,CCL19 and CCL22 detected by ELISA. Proliferation of various CIKs tested via 3H-TdR. Killing activities of k-ras-DCCIKs and CTLs examined with 125IUdR. *Results*. CD3^+^CD56^+^ and CD3^+^CD8^+^ were highly expressed by K-ras-DCCIKs. In its supernatant, IFN-*γ*, IL-12, CCL19 and CCL22 were significantly higher than those in DCCIK and CIK. The killing rate of K-ras-DCCIK was greater than those of CIK and CTL. CTL induced by K-ras-DCs only inhibited the PANC-1 cells. *Conclusions*. The k-ras-DC can enhance CIK's proliferation and increase the killing effect on pancreatic cancer cell. The CTLs induced by K-ras-DC can only inhibit PANC-1 cells. In this study, K-ras-DCCIKs also show the specific inhibition to PANC-1 cells, their tumor suppression is almost same with the CTLs, their total tumor inhibitory efficiency is higher than that of the CTLs.

## 1. Introduction

The incidence of pancreatic cancer has shown a clear uptrend [1], and the prognosis in last 20 years has not yet improved [2]. Current immunotherapies for pancreatic cancer mainly include active specific immunotherapy, monoclonal antibody-directed therapy, cytokine therapy, and adoptive cellular immunotherapy [3]. The aim of this study is to investigate the role of the cytokine-induced killer cells (CIKs) cocultured with dendritic cells (DCs) and pulsed with K-ras (12-Val) mutant peptide in the killing of pancreatic cancer cell lines, PANC-1 and SW1990, both in vivo and in vitro.

Dendritic cell (DC) is an antigen-presenting cell, whose function is strongest in the body. They play a role as the bridge and the pivot in the interaction of tumor cells and T lymphocytes [4]. The killer cells induced by cytokines IFN-*γ* and IL-2 and anti-CD3 monoclonal antibody (cytokine-induced killer, CIK) non-MHC-restrictive cytotoxic T lymphocytes, which kills tumor cells via recognition to a series of related ligands expressed in tumor surface [5, 6]. So far, these cells are considered to have the fastest proliferation, the strongest tumor cytotoxicity, and the most extensive range of tumor killing. Hence, they are the first choice for the adoptive immunotherapy of tumors [7]. Cocultivation of CIK and DC pulsed with K-ras (12-Val) protein peptide, which contains a specific mutation site, can increase the existence of antigen-specific CTL subsets and DC-induced specific CTL activity. Meanwhile, strengthening CIK cell proliferation can further be expected to improve the scope and effects of antitumor immunotherapy. As of now, the research on synergy therapy for pancreatic cancer with the K-ras antigen-allergized DC and CIK has not yet been reported.

## 2. Materials and Methods

### 2.1. Materials

rhIL-2 and GM-CSF were purchased from R&D Inc. (USA). IL-4, TNF-4, and IFN-*γ* were acquired from Peprotech Inc.. Fetal bovine serum (FBS) and cell medium RPMI1640 were sourced from Sigma-Aldrich Co, Ltd (USA). Lymphocyte separation medium Ficoll and normal human AB serum were purchased from TBD Inc. (Tianjin, China). Mouse anti-human CD3 (FITC labelled) monoclonal antibody, mouse anti-human CD56 (PE labelled) monoclonal antibody, mouse anti-human CD8 (PE labelled) monoclonal antibody, and mouse anti-human CD3 (unlabelled) monoclonal antibody were bought from eBioscience Co, Ltd. Mouse anti-human CD80-PE monoclonal antibody, mouse anti-human CD83-PE monoclonal antibody, mouse anti-human CD86-PE monoclonal antibody, mouse anti-human CD40-FITC monoclonal antibody, and mouse anti-human CD1a-FITC monoclonal antibody were all products of Immunotech Co, Ltd (France). CCL19 and CCL22 ELISA kits were from ADL Inc.. Mouse anti-human Fascin-1 monoclonal antibody and goat anti-mouse IgG secondary antibody were from Santa Cruz Co, Ltd. Cell strains, PANC-1 and SW1990, are available from ATCC. K-ras mutant epitope peptide KLVVVGAVGVGKSALTC was synthesized by SBS Genetech., Ltd.. Female nude mice (BALB/c, 5–8 weeks of age) raised under SPF circumstance were purchased from the Shanghai Laboratory Animal Center, Chinese Academy of Sciences.

### 2.2. Preparation of DCs and CTLs

50 mL of peripheral blood was sterilely collected from a healthy adult volunteer. Peripheral blood mononuclear cells (PBMCs) were then obtained by lymphocyte separation medium, washed twice with RPMI1640, then diluted to 2 × 10^6^/mL with RPMI1640 containing 10% (V/V) human AB serum. Subsequently, these cells were transferred into culture flasks and cultured for 1-2 hours. Nonadherent cells were harvested as the progenitor of cytokine-induced killer (CIK). The remaining adherent cells were cultured by adding DC medium (containing 0.2 mg/L GM-CSF, 1000 U/mL rhIL-4), and exchanged half amount of DC medium in the next day until 7 days. K-ras mutant epitope peptide was then added into the culture on the 7th day. After 24 hours of cultivation, the culture was induced by adding TNF-*α* (10 ng/mL) over the following 2 days. Lymphocytes at the final density of 2 × 10^5^/well were then mixed with the k-ras antigen-pulsed DC at 2 × 10^4^/well in 96-well plate, respectively. Under 37°C and 5% CO_2_, CTL cells were cultured for 5 days for induction by the specific antigen after which it was ready for use [8].

### 2.3. CIK Cell Induction and Proliferation

The density of the harvested nonadherent cells was adjusted to 1 × 10^6^/mL with PRMI1640 medium. After adding IFN-*γ* 1000 U/mL, the culture was cultivated under the condition of 37°C and 5% CO_2_ for 24 hours, when CD3 monoclonal antibody (50 ng/mL) and rhIL-2 (1000 u) was added. Subsequently, these cells exchanged half the amount of medium every three days and supplemented CD3 monoclonal antibody and rhIL-2 [9].

### 2.4. Culture of DCCIKs and Detection for their Cytokine and Proliferation Activity

In 96-well plates, CIKs with density 2 × 10^5^/well were mixed with the antigen-unpulsed DCs and K-ras peptide antigen-allergized DCs, which had been induced and cultured for 9 days, at the density of 2 × 10^4^/well. The cell mixtures were then cultured with CIK medium under the condition of 37°C and 5% CO_2_ for 5 days. After adding ^3^H-TdR (37 kBq/well), these cells were then cultured for another 12 hours. After 12 hours, the cell mixtures were collected and examined by verifying their cpm values with a liquid scintillation counter and by counting their stimulation index (SI): SI = (cpm of experimental group − cpm of background)/(cpm of control group − cpm of background). The proliferation of CIKs, DCCIKs, and K-ras-DCCIKs was observed. Moreover, IL-12 and IFN-*γ* in the supernatants of the cells cultured for 14 days were tested by ELISA.

### 2.5. Morphologic Observation and Cellular Phenotype Analysis of DCs and DCCIKs

Morphological changes of DCs and DCCIKs were observed by scanning and transmission electron microscopy after which, the DCs cultured for 7 days and the K-ras pulsed DCs cultured for 9 days were harvested. Using FACS, their phenotype molecules, CD1a, CD80, CD83, CD86, and HLA-DR, were measured and recorded. Afterward, the K-ras-DCs that were originally cultured for 9 days were co-cultivated with CIKs for 5 days. Subsequently, CIKs, DCCIKs, and K-ras-DCCIKs were collected at the 14th day of the cultivation, and the expression of surface markers, CD3, CD3^+^CD56^+^, and CD3^+^CD8^+^, was examined and recorded.

### 2.6. Detection of CCL19, CCL22, and Fascin-1 of k-Ras-DCCIKs

CIK cocultured with DC and DC pulsed with K-ras peptide at day 9th, the supernatants of the CIK, DCCIK and K-ras-DCCIK were collected at time points of preloading, 6 hours, 12 hours, 24 hours, and 48 hours, respectively. The CCL19 and CCL22 contents (absorbance) in the supernatants were tested separately by ELISA, three repeats for each group. Furthermore, CIKs, DCCIKs, and K-ras-DCCIKs that had been cultured for 14 days were harvested for protein extraction. Fascin-1 protein samples of each group were separated by SDS-PAGE and detected by western blot. *β*-actin was used as an internal reference. Mouse anti-human Fascin-1 monoclonal antibody was used as primary antibody (1 : 3000), and goat anti-mouse IgG polyclonal antibody was used as secondary antibody (1 : 5000).

### 2.7. Killing Activity of Different CIKs and CTLs to PANC-1 [10] and SW1990 Pancreatic Cancer Cells

The K-ras-DCCIKs, DCCIKs, CIKs, and CTLs cultured for 14 days were used as effector cells and PANC-1 and SW1990 as target cells. 1 × 10^6^ tumor cells in log phase were collected and added 5 *μ*Ci ^125^I-UdR and final concentration of 5 mol 5-fluorouracil. The cells were incubated in suspension culture under the condition of 37°C and 5% CO_2_ for 2 hours. After washing three times with IMDM medium to eliminate the unlabelled ^125^I-UdR, the tumor cells were counted with *γ*-counter. Only the cells that had average labelling yield above 1 cpm were used as target cells. Then, the target cells were adjusted with IMDM medium containing 10% FCS to the density of 5 × 10^5^/mL to be ready for use. In accordance with different effector-target ratio (1 : 6.25, 1 : 12.5, 1 : 25, 1 : 50), the effector cells were mixed with the target cells and supplemented with medium to yield one milliliter (three repeats). Meanwhile, the control group of target cells was used to test the spontaneous release rate. After centrifugation of 1000 r/min for 3 minutes, the effector-target cell mixtures were cultured for 12 more hours. Finally, the cell mixtures were centrifugated at 2000 r/min for 5 minutes and tested for their cpm values. The cytotoxic activity is shown with ^125^I-UdR release percentage, which is calculated according to the following formula: ^125^I-UdR release percentage = (cpm value of experimental group − cpm value of spontaneous release group)/(cpm value of maximum release − cpm value of spontaneous release group).

### 2.8. Animal Experiment

PANC-1 and SW1990 in log phase were prepared to 1 × 10^7^/mL cell suspension. Every female BALB/c nude mice of 5–8 weeks old were subcutaneously inoculated in their backs with 0.2 mL of the suspension to build the tumor-bearing mouse model. At the 10th day after inoculation, the mice were randomly divided into five groups (10 mice per group). Experimental group (I) Group k-ras-DCCIK, k-ras-DCCIKs, were used for every injection. (II) Group DCCIK, DCCIKs were used. (III) Group CIK, CIKs were used. (IV) Group CTL, CTLs induced with k-ras-DCs, were used. (V) Group saline control, saline water was used. 2 × 10^6^ CTLs and various CIKs were injected intratumor every two days, respectively, ten injections in total. Before every injection, the long diameter (*L*) and the short diameter (*S*) of the tumors were measured. And tumor sizes were estimated by formula: *V* = (*L* × *S* ^2^)/2. The final survival time of each group was observed.

### 2.9. Statistical Analysis

The statistic software SPSS 16.0 was used for data analysis, and Lab-wiok4.6 was used for analysis of western blot results. Measurement data were indicated with mean ± standard deviation. And the original data were tested via homogeneity of variance, and then used for *t*-test and variance analysis. The differences were deemed to show statistical significance when *P* < 0.05. And the final statistic values of different samples are the gray value ratios of the samples and their relevant internal references.

## 3. Results

### 3.1. Morphological Observation of DC and CIK

#### 3.1.1. Morphological Observation of K-ras (12-Val) Mutant Peptide-Pulsed DC

**Figure 1:**
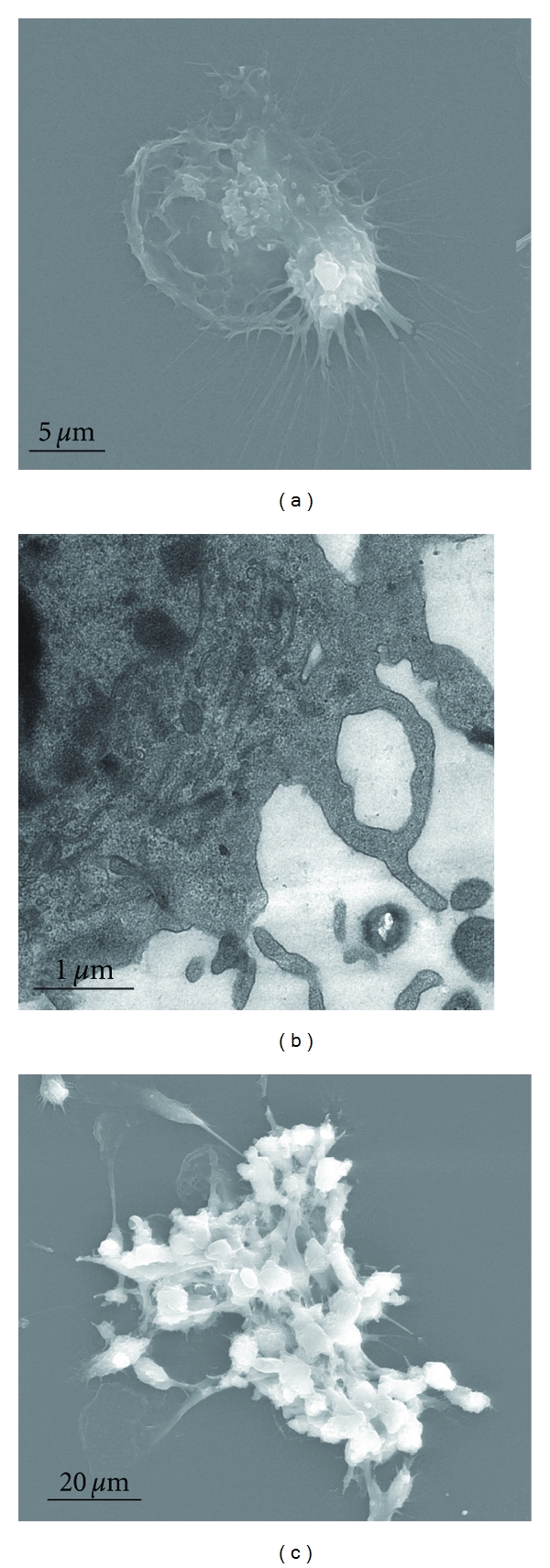
The morphological changes of k-ras pulsed DC and K-ras-DCCIK under scanning and transmission electron microscopy (experiment 2.5). DCs pulsed with K-ras mutant peptide show a larger soma, and plenty of dendritic bulges on their surfaces (Figure 1(a)). Under transmission electron microscopy, DCs show irregular-shape, large, and long dendritic bulges on their surfaces. In the DCs, organelles are abundant, and many mitochondria and rough endoplasmic reticulum can be seen, but less lysosome are present (Figure 1(b)). Coculture of DCs and CIKs for 14 days; under scanning electron microscopy, cells aggregated together to form many cell masses, on the surfaces of which there were plenty of dendritic bulges (Figure 1(c)).

After being pulsed with K-ras (12-Val) mutant peptide, DCs showed larger soma with plenty of dendritic bulges on their surfaces (Figure 1(a)) under scanning microscopy. The DCs also showed irregular shape. From the microscopy, large and long dendritic bulges and small ones were observed on the surface. In the DCs, organelles are abundant. Many mitochondria and rough endoplasmic reticulum were present in the image, though less lysosomes were observed (Figure 1(b)).

#### 3.1.2. Morphological Observation of CIK and DCCIK

Under microscopy, CIKs showed cluster-like growth. And after 3 days of incubation, CIKs' cell masses gradually multiplied and became larger. On the 7th day, the cells began to look rounded with regular shapes. After DCs and CIKs were cocultured for 14 days, the cells aggregated together to form many cell masses, on the surfaces of which there were plenty of dendritic bulges (Figure 1(c)).

### 3.2. Cellular Phenotype Detection of DC, CIK, and DCCIK

The expression levels of CD1a, CD80, CD83, and HLA-DR of K-ras-DC were higher than those of the unpulsed DC group (*P* < 0.05). However, no significant difference was observed with tCD86 expressions among the groups (*P* > 0.05) (Table 1.). This demonstrated that the dendritic cells can express the mature surface molecules after antigen allergization. After cocultivation, the K-ras-DCCIK population can express CD3^+^CD8^+^ and CD3^+^CD56^+^ at levels which were significantly higher than those of the unpulsed DCCIK group and the CIK group (*P* < 0.05) (Table 2).

### 3.3. ELISA Test for Chemokine, CCL19 and CCL22, and Western Blot Analysis for Cytoskeletal Protein, Fascin-1

**Figure 2:**
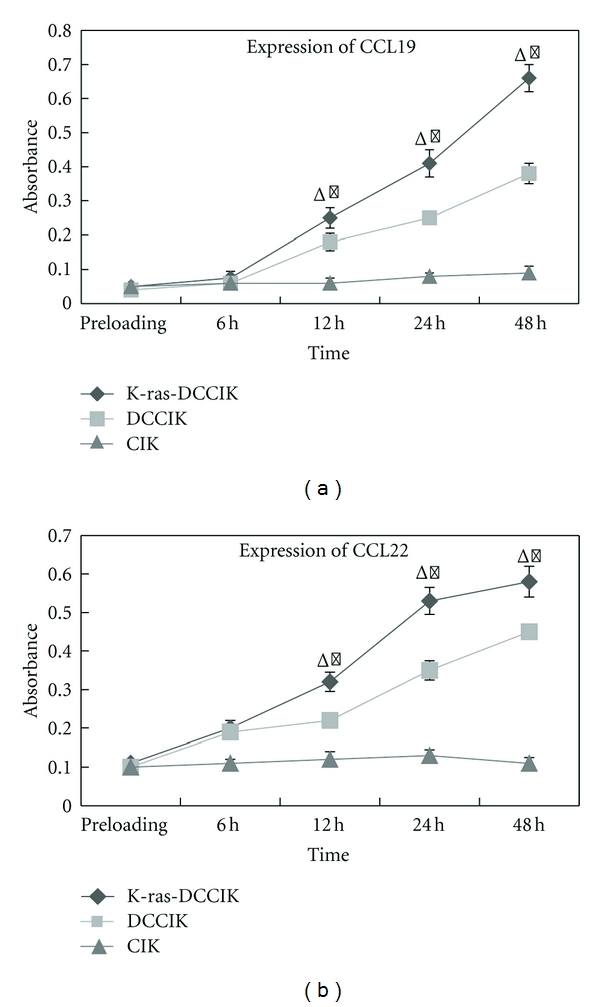
The absorbances of CCL19 and CCL22 at different time points (experiment 2.6). The expression of CCL19 and CCL22 in group K-ras-DCCIK and DCCIK showed uptrend with time and were higher than those of group CIK except for preloading and the 6-hour point. K-ras-DCCIK DCCIK versus CIK after 12 h, *P < 0.01. K-ras-DCCIK versus DCCIK after 12 h, *P* < 0.05. But the expression in group CIK showed no apparent increase (Figures 2(a) and 2(b)).

The CCL19 and CCL22 expression levels in the culture supernatants of group K-ras-DCCIK and group DCCIK were universally higher than those of group CIK except for preloading and the first 6-hour point. Moreover, the CCL19 and CCL22 levels in group K-ras-DCCIK and group DCCIK also showed uptrend with time. After testing at 12 hours, their levels increased more significantly (*P* < 0.01). While at the same time, CCL19 and CCL22 expression levels in group CIK showed no apparent increase. Finally, the comparison of the chemokine expression between group K-ras-DCCIK and group DCCIK also has statistical difference (*P* < 0.05) (Figures 2(a) and 2(b)).

#### 3.3.1. Western Blot Analysis for Cytoskeletal Protein, Fascin-1

**Figure 3:**
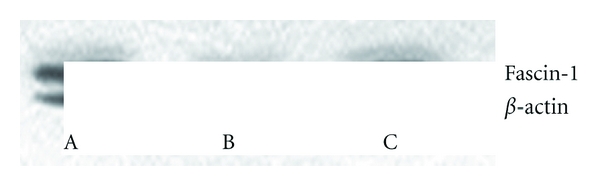
The fascin-1 and *β*-actin protein expression of DCCIK CIK and K-ras-DCCIK by western blot (experiment 2.6). A, B, and C demonstrate the fascin-1 expression in DCCIK, CIK, and K-ras-DCCIK cultured for 14 days. Compared with group DCCIK and CIK, fascin-1 in K-ras-DCCIK was increasing significantly (*P* < 0.01). DCCIK versus CIK has statistical significance (*P* < 0.05). The gray values of the reference protein bands are almost equal. It was shown that *β*-actin can be stably expressed in the cells.

The result is shown in Figures 3(a), 3(b), and 3(c), which demonstrates the fascin-1 expressions in DCCIK, CIK, and K-ras-DCCIK (cultured for 14 days). After protein bands analysis with Lab-wiok4.6, it is shown that the expression of the cytoskeletal protein, fascin-1, in K-ras-DCCIK had increased significantly. Compared with group DCCIK and group CIK, the differences showed statistical significance (*P* < 0.01). Furthermore, the comparison between DCCIK and CIK shows that the difference was also significant (*P* < 0.05). The gray values of the reference protein bands are almost equal. This proved that *β*-actin can be stably expressed in the cells. The results demonstrated that K-ras mutant antigen peptide can facilitate the migration activity of DCCIKs.

### 3.4. Proliferation Activity Test of CIK and DCCIK

**Figure 4:**
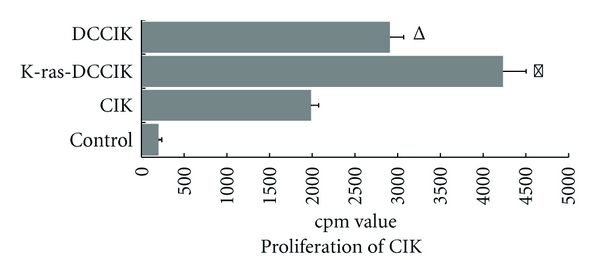
The proliferation activity of CIK cultured with K-ras antigen-pulsed DC (experiment 2.3, 2.4). When cultured for 14 days, proliferation capacity of K-ras-DCCIK was remarkably greater than other groups (*P < 0.01). And DCCIK proliferation was also greater than CIK (^Δ^ *P* < 0.05). It is proved that K-ras-DC can stimulate the proliferation of CIK effectively.

CIKs began proliferating from the third day of culture, and the cell proliferation sped up on the sixth day with the cell population increasing noticeably. When cultured for 14 days, proliferation capacity of K-ras-DCCIK was remarkably greater than that of other groups (*P* < 0.01). DCCIK proliferation was also greater than CIK (*P* < 0.05) (Figure 4). This showed that K-ras-DC can stimulate the proliferation of CIK effectively. After being allergized by peptide antigen, the DC's ascending secretion of IFN-*γ* and IL-12 further stimulated the CIK's proliferation.

### 3.5. Detection of Cytokine, IL-12 and IFN-*γ*

**Figure 5:**
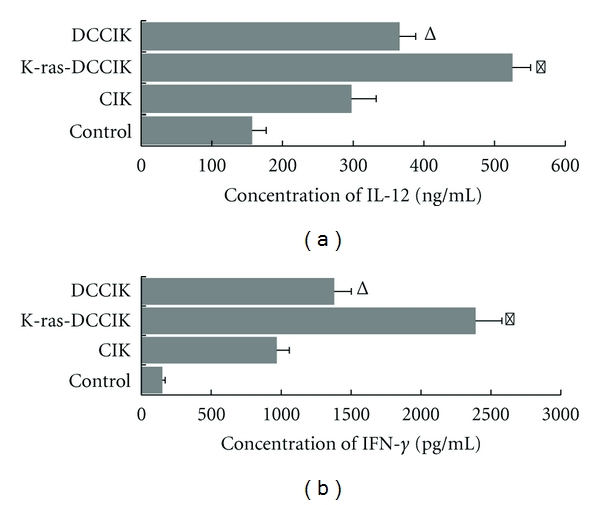
The production of IL-12 and IFN-*γ* of CIK cultured with K-ras antigen-pulsed DC (experiment 2.4). In the supernatant of group K-ras-DCCIK cultured for 14 days, IFN-*γ* and IL-12 levels were highest, K-ras-DCCIK versus DCCIK and CIK (*P < 0.01). And levels in group DCCIK were also higher than those of CIK (^Δ^ *P* < 0.05). It is demonstrated that after coculture of CIKs and k-ras-DCs, IFN-*γ* and IL-12 levels in the cell supernatants can be apparently increased.

In the supernatant of group K-ras-DCCIK cultured for 14 days, IFN-*γ* and IL-12 levels were higher than those of group CIK and group DCCIK (*P* < 0.01). And IFN-*γ* and IL-12 levels in the supernatant of group DCCIK were also higher than those of group CIK (*P* < 0.05. After coculture of CIKs and DCs, IFN-*γ* and IL-12 levels in the cell supernatants can be increased. Furthermore, the antineoplastic activity of the specific antigen-pulsed DCCIK became stronger (Figure 5).

### 3.6. Detection for Killing Activity of CIK and CTL to PANC-1 and SW1990 Pancreatic Cancer Cell In Vitro

**Figure 6:**
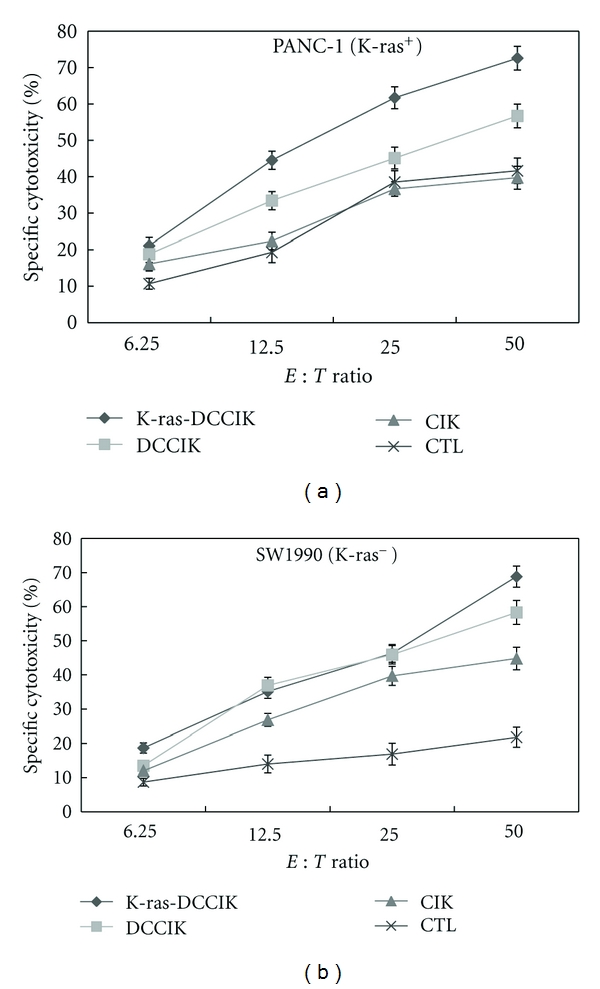
The cytotoxicity of K-ras-DCCIK, DCCIK, CIK, and CTL induced by K-ras pulsed DC against PANC-1 (K-ras^+^) and SW1990 (K-ras^−^) cells in vitro (experiment 2.7). The K-ras-DCCIKs, DCCIKs, CIKs, and CTLs induced by K-ras pulsed DCs used as effector cells. PANC-1 and SW1990 used as target cells. The killing effects on PANC-1 showed that group K-ras-DCCIK exceeded group CIK and group CTL remarkably (*P* < 0.01). However, there was no difference between group CIK and CTL (*P* > 0.05), (Figure 6(a)). The different killing effects on SW1990 demonstrated K-ras-DCCIKs, DCCIKs, and CIKs; all showed their killing effects on SW1990 cells and are higher than CTL (*P* < 0.01), (Figure 6(b)).

The K-ras-DCCIKs, DCCIKs, CIKs, and CTLs induced by K-ras pulsed DCs were used as effector cells, and the pancreatic cancer cell strains, PANC-1 and SW1990, were used as target cells. The different killing effects of the CIK groups on PANC-1 showed that the killing rate of group K-ras-DCCIK was greatest and significantly exceeded group CIK and group CTL (*P* < 0.01). However, there was no difference between group CIK and group CTL (*P* > 0.05). After increasing the effector-target ratio, the killing rates of the effector cells against the pancreatic cancer cells in all the groups also became higher (Figure 6(a)). As the different killing effects of the CIKs groups on SW1990 demonstrated K-ras-DCCIKs, DCCIKs, and CIKs, all showed their killing effects on SW1990 cells, and their killing rates are higher than group CTL (*P* < 0.01). But the comparison of the killing effect between group K-ras-DCCIK and group DCCIK showed no statistical significance (*P* > 0.05) (Figure 6(b)).

**Figure 7:**
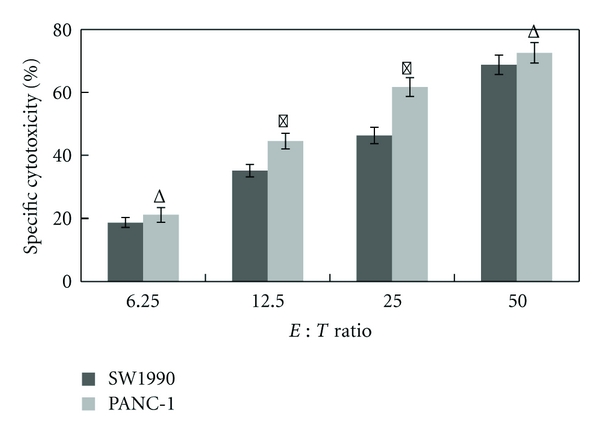
The cytotoxicity of K-ras-DCCIK against PANC-1 (K-ras^+^) and SW1990 (K-ras^−^) cells at different *E* : *T* ratio in vitro (experiment 2.7). It was found that when effector-target ratio reached 1 : 12.5 and 1 : 25, K-ras-DCCIKs' inhibition to PANC-1 was stronger than that to SW1990 (*P < 0.05). However, *E* : *T* ratio at 1 : 6.25 and 1 : 50, K-ras-DCCIKs' inhibition to these two cells showed no statistical difference (^Δ^ *P* > 0.05). It is demonstrated that CIK cells have a potential to uptake tumor antigens, and further, to produce specific killing effect on PANC-1, there might be some antigen-specific CTL cell subsets existing in K-ras-DCCIKs.

By testing the in vitro killing inhibitions of K-ras-DCCIKs to PANC-1 (K-ras^+^) and SW1990 (K-ras^−^), it was found that, when effector-target ratio reached 1 : 12.5 and 1 : 25, K-ras-DCCIKs' inhibition to PANC-1 was stronger than that to SW1990 (*P* < 0.05). However, effector-target ratio increased to 1 : 50; K-ras-DCCIKs' inhibition to these two cells showed no statistical difference (*P* > 0.05) (Figure 7).

### 3.7. The Effects of Various CIKs and CTL on Survival Time of Tumor-Bearing Nude Mice Loading PANC-1 and SW1990 Pancreatic Cancer Cells

**Figure 8:**
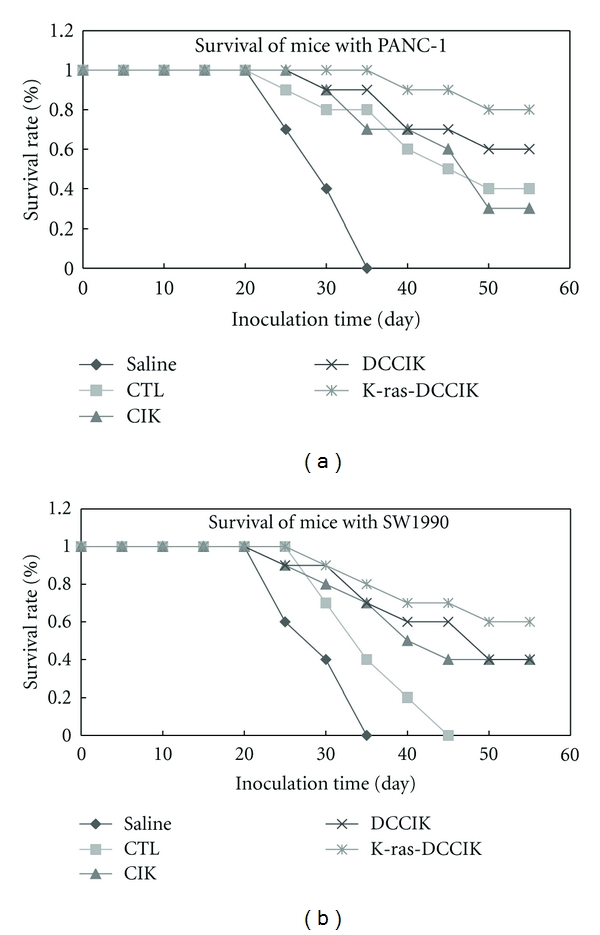
Survival of nude mice inoculated subcutaneously in the back with PANC-1 (K-ras^+^) and SW1990(K-ras^−^) cells after immunotherapy with CTLs and different CIKs (experiment 2.8) concerning the effects on the survival time of PANC-1 (K-ras^+^) tumor-bearing mice (Figure 8(a)), the survival time of group K-ras-DCCIK was prolonged remarkably, compared with other groups (*P* < 0.01). No statistical difference was found among the group DCCIK, CIK and CTL (*P* > 0.05). It is demonstrated that the k-ras-DC induced CTL can inhibit PANC-1. Meanwhile, K-ras-DCCIK can produce specific and immediate killing effect on PANC-1. Concerning the effects on the survival time of SW1900 (K-ras^−^) tumor-bearing mice (Figure 8(b)), the survival time of group K-ras-DCCIK, DCCIK and CIK was elongated dramatically. Compared with group CTL (*P* < 0.01). The CIK groups possess the direct inhibition to SW1900. The CTL induced by K-ras-DC showed the lower inhibition to K-ras mutation negative cell, SW1900. Thus, the survival time of the SW1900 tumor-bearing mice was not extended.

2 × 10^6^ cells of CIK groups and k-ras-DC-induced CTL group were injected intratumorly into the tumor-bearing nude mice, and their effects on the mice survival time were investigated. Concerning the effects on the survival time of PANC-1 (K-ras^+^) tumor-bearing mice (Figure 8(a)), the survival time of group K-ras-DCCIK was prolonged remarkably. There is significant difference in comparison with other groups (*P* < 0.01). In group DCCIK, group CIK, and group CTL, the mice survival times were extended correspondingly. But there were no statistical differences among the groups (*P* > 0.05). It is demonstrated that the DC-induced specific CTL can inhibit PANC-1. Meanwhile, K-ras-DCCIK can produce specific and immediate killing effect on PANC-1. This will lead to prolonged survival time. Concerning the effects on the survival time of SW1900 (K-ras^−^) tumor-bearing mice (Figure 8(b)), the survival time of group K-ras-DCCIK, group DCCIK, and group CIK was elongated dramatically. Also, compared with group CTL, the difference has statistical significance (*P* < 0.01). However, there were still no significant differences among the groups (*P* > 0.05). It is shown that to varying degrees, the CIK groups possess the direct inhibition to SW1900. In contrast, the specific CTL induced by K-ras mutant peptide-pulsed DC showed the lower inhibition to K-ras mutation-negative cell, SW1900. In group CTL, the survival time of the SW1900 tumor-bearing mice was not extended.

## 4. Discussions

Tumorigenesis is a sustained process of gene mutations. Almoguera et al. [11] first reported the point mutation of K-ras gene in pancreatic cancer sufferers. Since, it had been investigated that, in 85%–95% of pancreatic cancer patients, K-ras gene mutations occurred and almost all the mutations happened at the 12th codon. Hence, the 12th mutational site of K-ras protein can be used as a potential site for gene immunotherapy of pancreatic cancer [12, 13]. Nakada et al. [14] and others had used the antisense oligonucleotides for K-ras gene mutations to transfect the pancreatic cancer cell, PANC-1. This treatment can inhibit the mRNA expression of K-ras gene and the synthesis of ras protein. Thus, it can suppress the growth of pancreatic cancer cell and facilitate the apoptosis of cancer cells. He et al. [15] and others had tried to use K-ras mutated peptide to modify DCs in order to activate T cells. It had been found that DCs can present K-ras mutated sites effectively. In this study, we used K-ras mutated peptide to modify DCs. After coculture with the modified DCs, CIKs showed immediate and specific inhibition to pancreatic cancer cells in vitro and in vivo.

CIKs is a cell population obtained from human peripheral blood mononuclear cells stimulated with IFN-*γ*, IL-2, and CD3 monoclonal antibody (OKT3). They can express the surface markers of T cells and NK cells, CD3^+^CD56^+^ [16]. For now, CIK is known to have the fastest proliferation, the strongest tumor cytotoxicity, and the most extensive range of tumor killing [17]. CIKs' killing action to tumors is via recognition of a series of associated ligands on tumor cell surfaces, though not only depending upon one antigen [5]. CIKs can both directly inhibit tumor cells and regulate immune system of body to kill tumor cells indirectly [18]. Therefore, they can suppress the tumor's growth and recurrence by immediate inhibition to tumor cells and improve the immunity of patients for long-term effect [19]. CIKs seldom arouse graft-versus-host disease (GVHD) and are safe and effective for patients who had developed drug resistance. Coculture of CIKs with DCs can increase their proliferation activity and cytotoxicity [20]. At present, the mechanism of why DCs can enhance CIKs' killing activity is still unclear. It has been speculated that the strengthened tumor-killing effect of DCCIKs may be associated with upregulation of cytokines, such as IL-12 and IFN-*γ*, in DCCIKs' supernatant and with high expression of CD3^+^CD56^+^ double positive cells as well [21]. In this study, by testing the expressions of DCs' surface molecules, the results show that K-ras mutated peptide can promote DCs' mature and facilitate effective presentation of specific antigens [22]. IFN-*γ* and IL-12 levels of group K-ras-DCCIK are highest, superior to those of group DCCIK and group CIK remarkably (*P* < 0.01). Moreover, after co-cultivation, K-ras-DCCIKs highly expressed CD3^+^CD8^+^ and CD3^+^CD56^+^, exceeding those of group CIK significantly (*P* < 0.05).

The chemokine, CCL19, is expressed in secondary lymphoid organs and thymus. It can urge DCs to migrate from peripheral region to T-cell accumulation area in lymphoid organs and induce Th1 and T cells to make an immune response [23–25]. CCL22 is expressed in the spleen, peripheral blood T cells, NK cells, and so on. [26]. In the supernatant of the monocyte-derived DCs, intact CCL22 become highly expressed and produce intense chemotaxis for DCs [27, 28]. In ELISA test for CCL19 and CCL22 of various CIK groups, it was founded that k-ras-DCs can apparently enhance CIKs' migration activity and can improve their migration capacity towards tumor cells. These actions provide some necessary conditions for increasing killing activity and suppressing tumor growth. In immune system, Fascin-1 protein is only expressed in the mature DCs and related to DCs' movements [29, 30]. The research indicated that the secretory volume of k-ras-DCCIKs' cytoskeletal protein Fascin-1 was increased remarkably. Compared with group DCCIK and group CIK, the increasing has dramatically statistical significance (*P* < 0.01). It was demonstrated that K-ras mutated peptide can induce DCs' mature and enhance k-ras-DCCIKs' migrating capacity.

k-ras-DCCIKs' proliferation capacity is significantly higher than that of other groups (*P* < 0.01). It can be seen that k-ras-DC can effectively stimulate CIKs' proliferation. Moreover, the increased secretion of IFN-*γ* and IL-12 further irritated CIKs' proliferation. Marten and so forth [31] used CA19-9 antigen-pulsed mature DCs and antigen peptide-untreated DCs to coculture with CIKs. In contrast with untreated DCs group, the former showed increased killing activity. It was prompted that, in the CIKs cocultured with CA19-9 pulsed DCs, there were existing antigen-specific CTL subsets. In our in vitro experiment, the killing activity of K-ras-DCCIK group to PANC-1 cells was also superior to those of CIK group and k-ras-DC-induced CTL group (*P* < 0.01). It indicates that, via K-ras pulsing, the DCs further enhanced CIKs' killing activity. Compared with k-ras-DC-induced specific CTLs, CIKs have almost identical killing efficiency (*P* > 0.05). The specific CTLs have obvious inhibition to K-ras^+^ PANC-1, while K-ras-DCCIKs have immediate PANC-1 killing effect, which is specific and more remarkable so as to make the mice's survival period significantly prolonged. All of the K-ras-DCCIKs, DCCIKs, and CIKs showed inhibitions to SW1990 cells. But k-ras-DC-induced specific CTLs present the weakened suppression towards SW1990 cells. Compared with DCCIKs' inhibition, there is a significant statistical difference (*P* < 0.01). It was shown that, to varying degrees, the CIK groups possess the direct inhibition to SW1900. In contrast, the specific CTLs showed the lower inhibition to K-ras mutation-negative cell, SW1900. In group CTL, the survival time of the SW1900 tumor-bearing mice was not extended. In this study, by co-culturing tumor antigen-pulsed DCs with CIKs, we obtained the k-ras-DCCIKs, which have more prominent oncotherapy effect and more powerful tumor inhibition than CIKs and show a favourable application prospect.

PANC-1 and SW1990 cells were inhibited with k-ras-DCCIK separately. The results show that, when effector-target ratio reached 1 : 12.5 and 1 : 25, K-ras-DCCIKs can produce specific inhibition to PANC-1. Further, the killing efficiencies towards these two pancreatic cancer cells have statistical difference (*P* < 0.05). However, increasing effector-target ratio, the difference of specific tumor inhibition for these two cells showed no statistical significance (*P* > 0.05). It is demonstrated that CIK cells have a potential to uptake tumor antigens and further to produce specific killing effect on cancer cells. In K-ras-DCCIKs, there might be some antigen-specific CTL cell subsets existing. Allergization for CIKs with tumor antigen-pulsed DCs can both exert non-MHC restrictive cytotoxicity of CIKs and activate MHC restrictive cytotoxicity mediated by antigen-pulsed DCs to strengthen the specific killing effect on specific target cells [32]. However, when effector-target ratio is high, it might be CIKs' strong tumor direct killing effect that covers their specific action. This phenomenon is to be further investigated in the future.

In conclusion, the results demonstrate that, after being pulsed with K-ras, DCs can enhance CIKs' proliferation and migration capacities, and can enhance killing activity against pancreatic cancer cells as well. Moreover, CIKs' enhanced killing activity may be associated with upregulation of IFN-*γ* and IL-12 in supernatants and high expression of double-positive cells CD3^+^CD8^+^ and CD3^+^CD56^+^. The antigen-allergized DCCIKs can produce in vitro killing activity specific to tumor cells. Their pertinence of tumor suppression is almost the same as with the specific CTLs, while their total tumor inhibitory efficiency is higher than the CTLs.

## Figures and Tables

**Table 1:** Surface marker of DCs induced by different antigens (%, *X* ± SD, *n* = 6).

Groups	CD1a	CD80	CD83	CD86	HLA-DR
DC	22.6 ± 3.6	49.4 ± 3.2	38.5 ± 4.6	72.6 ± 5.6	66.5 ± 4.6
DC(K-ras^+^ peptide,10 *μ*g/mL)	35.1 ± 4.3	62.2 ± 5.8	51.1 ± 4.9	74.4 ± 5.2	82.4 ± 4.4

Note: DC (K-ras^+^ peptide, 10 *μ*g/mL) versus DC, *P* < 0.05 except CD86.

**Table 2:** Surface marker of CIK and DCCIK induced by antigens (%, *X* ± SD, *n* = 6).

Groups	CD3	CD3^+^CD56^+^	CD3^+^CD8^+^
CIK	66.34 ± 4.54	34.18 ± 2.63	56.38 ± 4.87
DCCIK	71.4 ± 55.26	39.21 ± 3.12	54.23 ± 4.14
K-ras-DCCIK	87.53 ± 6.02	57.43 ± 4.34	68.65 ± 3.32

Note: DCCIK pulsed with K-ras peptides versus CIK and DCCIK, (*P* < 0.05).

## Abbreviations

CTLs: Cytotoxic T lymphocytes
DCs: Dendritic cells
GM-CSF: Granulo-macrophage-stimulating factor
IL: Interleukin
CIK: Cytokine-induced killer
DCCIKs: CIK cells cocultured with DCs
CD1a, CD80,
CD83, CD8,
and HLA-DR: DC surface markers
CD3^+^CD8^+^
and CD3^+^CD56^+^: CIK surface markers
Fascin-1: A kind of cytoskeletal protein
PANC-1: The pancreatic cancer cell line with point mutations of K-ras
SW1990: The pancreatic cancer cell line without point mutations of K-ras
SI: Stimulating index
TNF-*α*: Tumor necrosis factor
PBS: Phosphate-buffered saline
IFN-*γ*: Interferon
FCS: Fetal calf serum
ELISA: Enzyme-linked immunosorbent assay.
